# The role of self-efficacy and parental communication in the association between cyber victimization and depression among adolescents and young adults: a structural equation model

**DOI:** 10.1186/s12888-023-04841-6

**Published:** 2023-05-12

**Authors:** Chanda Maurya, T. Muhammad, Ayushi Das, Abdul Fathah, Preeti Dhillon

**Affiliations:** 1 Department of Survey Research & Data Analytics, International Institute for Population Sciences, Mumbai, India; 2 Department of Family & Generations, International Institute for Population Sciences, Mumbai, India; 3 International Institute for Population Sciences, Mumbai, India

**Keywords:** Cyber victimization, Depressive symptoms, Self-efficacy, Parental communication

## Abstract

**Background:**

With the rapid advancement and revolutionization of information and communication technologies, adolescents and young adults use smartphones, the internet, and social networking services more frequently, as a result, the problem of cyber-bullying sharply increases, and eventually it causes psychological issues and negative thoughts in the victims. This study aimed to examine the role of self-efficacy and parental communication in the relationship between cyber victimization and depression among adolescents and young adults in India.

**Methods:**

Secondary data analysis was performed on a cross-sectional dataset obtained from the Understanding the Lives of Adolescents and Young Adults (UDAYA) wave 2 survey. The sample included 16,292 adolescent and young adult boys and girls aged 12–23 years. Karl Pearson Correlation coefficient analysis was done to examine the correlation between outcome variable (depressive symptoms), mediator variables (self-efficacy and parental communication) and key explanatory variable (cyber victimization). Further, structural equation modeling technique was applied to examine the hypothesized pathways.

**Results:**

Adolescents and young adults being victims of cyber-bullying [β = 0.1357, p < 0.001] and those witnessed inter-parental violence were positively [β = 0.0026, p < 0.001] correlated with depressive symptoms. Self-efficacy and parental communication were negatively related to depressive symptoms among adolescents and young adults. There was a significant positive relationship between cyber victimization [β = 0.258, p < 0.001] and depressive symptoms. Cyber victimization was positively related to self-efficacy [β = 0.043, p < 0.001] among adolescents and young adults. Self-efficacy [β= -0.150, p < 0.001] and parental communication [β=-0.261, p < 0.001] reduced depressive symptoms among the participants.

**Conclusions:**

The findings suggest that adolescents and young adults who are victims of cyber-bully may have depressive symptoms and their mental health can be improved through the enhancement of self-efficacy and increased parental communication. Improved peer attitudes and familial support for empowering cyber victims should be taken into account while framing programs and interventions.

**Supplementary Information:**

The online version contains supplementary material available at 10.1186/s12888-023-04841-6.

## Background

Cyber-bully is an intentional, aggressive nature of behavior that takes place in electronic communication in order to bully a person. Cyber-bully is an important issue especially among adolescents which seems to be a common feature of interpersonal relationships. Approximately, 20–40% of adolescents have been victims of cyber-bully globally [1]. Besides, earlier research has shown that electronic aggression is an emerging public health concern and calls for special attention in terms of awareness programs and parental monitoring and communication for the prevention of cyber-bully and victimization [2]. Adolescents who are victims of cyber-bully can have a considerable impact on depression [3]. It is documented that cyber victimization is the main cause of depression among adolescents [1].

Early adulthood is the phase where individuals focus on developing emotional stability, deciding a career and finding intimacy. Adolescents who are victims of cyber-bully even at low levels may possess more depressive symptoms compared to others who are not exposed to cyber victimization and they are at excessive risk of future mental health problems [1, 4]. Studies suggest that cyber victimization and related mental health problems needs to be seriously approached with efficient strategies because cyber-bully victims possess a higher risk of depression and suicidal thoughts [19]. Growing body of evidence demonstrate that adolescents not only experience difficulties of mood disorders but also bear the risk of morbidity and mortality due to depression [5, 6].

Depressed people often possess low self-efficacy which refers to perceived potential of an individual to influence situational outcome, and lack of self-efficacy might lead an individual to depressed condition through a discrepancy in aspirations and perceived skills [7]. Self-efficacy has a significant relationship with quality of life and depression and it reduces the risk of depression and increase the quality of life [8, 9]. Since self-efficacy is negatively correlated with depression, enhancing the level of self-efficacy is a remarkable self-management intervention to improve and prevent depression [10]. Confidence and positive self-perceptions possess statistically significant arbitrate effects on the future relationship between self-efficacy and depressive symptoms [11, 12]. Similarly, family environment plays a pivotal role in shaping adolescent mental health. Parental communication was a protective element for depressive symptoms among all boys and girls [13]. The healthy communication may show an overall healthier relationship between parents and adolescents, which may act as a protective factor against the development of depressive symptoms. In order to deal with the depressive symptoms, both maternal and paternal perception of satisfactory communication is essential, but if the adolescent is satisfied with communication level with any one of the parents, it can settle accounts with the poor communication with the other parent [14].

Depressive symptoms among adolescents due to cyber-bully are associated with several other factors. There is a highly significant positive association between education level and depression [15]. Several studies have examined that adolescents who are exposed to experience inter parental violence have a higher chance of possessing depressive symptoms [16, 17]. Similarly, although internet delivers countless benefits; it also has negative consequences, and increased internet access among adolescents and children leads to cyber-bully at certain stages which causes depression among them [18, 19]. Depressive symptoms may vary according to place of residence. Due to various reasons, generally, rural residents possess higher depressive symptoms compared to urban residents and studies from India and other developing countries found that the prevalence of depression among younger population is significantly higher among residents in rural areas than in urban areas [20, 21]. Furthermore, a number of epidemiologic studies have shown that depressive symptoms are relatively high in women compared to men [22]. In India, women’s lower socio-economic status and restricted access to resources again leads to higher prevalence of depression [23, 24].

Depression among adolescents and young adults may impose a health burden on both their family and society. Therefore, it is important to understand the newly emerging factors associated with depression in this population and frame policies that may help reduce depressive symptoms among adolescents and young adults, especially in low-resource countries like India. On the other hand, with the rapid advancement and revolutionization of information and communication technologies, adolescents and young adults use smartphones, the internet, and social networking services more frequently, as a result, the problem of cyber-bullying sharply increases, and eventually it causes psychological issues and negative thoughts in the victims [25–27]. To fill in the knowledge gaps, this study aimed to examine the role of self-efficacy and parental communication in the relationship between cyber-bully victimization and depression symptoms among adolescents and young adults in India. Such an investigation can help policymakers and health practitioners develop policies and implement strategies with regard to addressing adolescents’ and young adults’ mental health issues. The study inspects such associations after controlling for several confounding variables such as age, educational level, inter-parental violence, internet access, paid work, mother’s education, wealth index, caste, religion and place of residence.

## Materials and methods

### Data

Secondary data analysis was performed on a cross-sectional dataset obtained from the Understanding the Lives of Adolescents and Young Adults (UDAYA) wave 2 survey. The survey was conducted in the two Indian states Uttar Pradesh and Bihar, in 2015-16 and 2018-19 by Population Council under the leadership of Government of India’s Ministry, the Ministry of Health and Family Welfare [28]. The UDAYA collected detailed information on family, community environment, media, assets acquired in adolescence, and quality of transitions to young adulthood indicators. Uttar Pradesh and Bihar’s sample sizes were 10,350 and 10,350 adolescents aged 10–19 years at wave 1, respectively. The sample for each sub-group of adolescents was determined at 920 younger boys, 2350 older boys, 630 younger girls, 3750 older girls, and 2700 married girls in both states. The survey adopted a multi-stage systematic sampling design to provide the estimates for states as a whole and for the urban and rural areas of the states. The detailed information on the sampling procedure and survey design was published elsewhere [28]. The ethical approval for the UDAYA survey was collected from the Institutional Review Board of the Population Council, New Delhi, India. Informed consent was collected in verbal and written forms from the respondents or their guardians during the survey. All the methods were carried out in accordance with relevant guidelines and regulations of the Human Subjects Protection. The compelling sample size for this study was 16,292 adolescent and young adult boys and girls aged 12–23 years.

### Outcome variable

The outcome variable was depressive symptoms among adolescents and young adults. Depressive symptoms were determined using the patient health questionnaire (PHQ-9). The respondents were asked about the symptoms of depression in the past two weeks. The nine questions included, (i) had trouble falling asleep or sleeping too much, (ii) feeling tired or having little energy, (iii) poor appetite or eating too much, (iv) trouble concentrating on things, (v) had little interest or pleasure in doing things, (vi) feeling down, depressed, or hopeless, (vii) feeling bad about yourself, (viii) been moving or speaking slowly, (ix) had thoughts that respondent would be better off dead [28]. All the questions were asked based on a Likert scale of four, from “not at all” to “nearly every day”. Further, a continuous variable with an alpha value 0.82 was created and higher the value indicates higher level of depressive symptoms [29].

### Exposure variable

#### Cyber victimization

Cyber victimization was measured using the two questions “Has anyone ever used a cell phone or text messaging to bother or harass you or to spread mean words or pictures about you?” and “Has anyone ever used the internet to bother or harass you or to spread mean words or pictures about you?” and categorized as “yes” if the respondent gave positive response from at least one question and “no” otherwise [27].

### Mediator variables

#### Self-efficacy

Self efficacy was assessed by using six questions (i) How sure are you that you could choose how to spend your free time?, (ii) How sure are you that you could participate in no-family/non-school related events/functions?, (iii) How sure are you that, you can choose to earn an income if you wish to?, (iv) How sure are you that you can talk freely to your parents/in-laws about your aspirations, for example, how far to study, whether to work or not, what you would like to become in the future etc?, (v) How sure are you that you can choose the type of clothing that you would like to wear? and (vi) How sure are you that you can play strong role in resolving family conflicts? [28]. All the questions were asked on a scale of five, i.e., 1 “not at all”, 2 “somewhat unsure”, 3 “neither sure/unsure, 4 “somewhat sure” and 5 “completely sure”. Continuous variable with alpha value 0.65 was taken into consideration. Higher value shows higher value of self-efficacy.

#### Parental communication

Parental communication was measured by using five questions about the parental interaction/relationship. The five questions included, (i) In the past one year, have you discussed about school performance with your mother or father? (ii) In the past one year, have you discussed about your friendship with your mother or father? (iii) In the past one year, have you discussed about being teased with your mother or father? (iv) In the past one year, have you discussed about menstruation to the girls and about the physical chances in body (e.g. voice change, facial hair growth etc.) to the boys with your mother or father? (v) In the past one year, have you discussed about how pregnancy occurs with your mother or father? [28]. All the above questions were recoded as 1 “yes” if the respondent had discussed about the above items either with mother/father or with both and 0 “no” otherwise. All the items were summed and a continuous variable (ranges 0–5) was created with an acceptable alpha value of 0.68. Higher value indicates higher value of parental communication.

### Covariates

Given existing evidence linking adolescents’ and young adults’ depression to multiple socio-demographic characteristics in particular Indian context [5, 21, 27, 30], we controlled for the effect of the following variables in the current analysis. Age group was recoded as 12–19 years representing “Adolescents” and 20–23 years representing “Young adults”. Sex of the respondent was coded as “Male” and “Female”. Educational level was recoded as “No education”, “Primary or secondary” and “Higher”. Inter-parental violence was assessed through the direct question “Has your father ever beaten your mother? For the positive response, it was coded as “Yes” and “No” otherwise. Internet access was recoded as “Yes” and “No”. Paid work was recoded as “Yes” and “No”. Mother’ education was recoded as “Illiterate” and “Literate”. Wealth index was recoded as “Poor”, “Middle” and “Rich”. Caste was recoded as “SC/ST” and “Non-SC/ST”. Religion was recoded as “Hindu” and “Non-Hindu”. Place of residence was recoded as “Urban” and “Rural”. State was coded as “Bihar” and “Uttar Pradesh”.

### Statistical analysis

Descriptive statistics was done to show the sample characteristics of the study population, bivariate analysis was done to examine the preliminary results. Independent t-tests were used to assess the gender difference in depressive symptoms according to the background variables. Karl Pearson Correlation coefficient analysis was done to examine the correlation between outcome variable (depressive symptoms), mediator variables (self-efficacy and parental communication) and key explanatory variable (cyber victimization). Further, for the estimation of covariance matrix, SEM (structural equation modeling) technique using the Maximum Likelihood estimation procedure was applied. Statistical package Stata 14 was used for the descriptive statistics and the SEM analysis. Three criteria are generally used in the evaluation of SEM, which include the model-fit indices, the statistical significance of the parameter estimated and the effect size and its direction.

Model fit was examined using guidelines (reference values) according to which good model fit is reached when chi-square value is low and non-significant; comparative fit index (CFI) values are 0.95 or more, and root mean square error of approximation (RMSEA) values are 0.05 or less (0.6–0.8 indicates a mediocre model fit). Chi-square difference testing and Akaike Information Criterion (AIC) was used to compare the models for the best fit whereby the lowest AIC indicated the best fitting model [31]. Statistically significant coefficients within the best fitting model were then examined for interpreting specific cyber victimization and depressive symptoms mediated by the self-efficacy and parental communication.

## Results

Sample characteristics of the study population are represented in the Table 1. About 84.2% of males and 95.8% of females were adolescents (aged 12–19 years). Nearly 62.2% of males and 55.1% of females had higher educational level and 2.1% of males and 12.3% of females were illiterate. Nearly 6.1% of males and 5.7% of females witnessed inter-parental violence. A higher percentage of males (73.9%) had access to internet while this percentage was low among females (33.6%). Nearly 44.7% of males and 23% of females were doing paid work. Nearly 70% of the males’ and 74.7% of the females’ mothers were illiterate. A higher percentage of males and females belonged to non-SC/ST caste and about 15.1% of males and 21% of females were non-Hindu. Most adolescents and young adults lived in rural areas (males-83.2% and females-84.5%).

**Table 1:** Sample characteristics of study population

Background variables	Male		Female	
	Sample	Percent	Sample	Percent
Age group				
Adolescents (12–19 years)	3,740	84.15	11,356	95.84
Young adults (20–23 years)	688	15.85	508	4.16
**Educational level**				
Never	111	2.05	1,588	12.27
Primary & middle	1,518	36.80	3,540	32.64
Higher	2,799	62.15	6,736	55.09
**Inter-parental violence**				
No	4,183	93.93	11,183	94.30
Yes	245	6.07	681	5.70
**Internet access**				
No	920	26.08	6,955	66.36
Yes	3,508	73.92	4,909	33.64
**Paid work**				
No	2,551	55.45	9,355	78.05
Yes	1,877	44.65	2,509	21.95
**Mother’s education**				
No	2,875	70.01	8,574	74.73
Yes	1,553	30.15	3,290	25.27
**Wealth index**				
Poor	1,113	31.31	3,385	31.28
Middle	908	22.00	2,490	21.76
Rich	2,407	46.44	5,989	46.96
**Caste**				
SC/ST	1,086	27.01	2,873	26.15
Other	3,342	72.66	8,991	73.85
**Religion**				
Hindu	3,729	84.93	9,300	79.03
Non-Hindu	699	15.07	2,564	20.97
**Place of residence**				
Urban	1,989	17.01	4,934	15.55
Rural	2,439	83.22	6,930	84.45
**State**				
Uttar Pradesh	2,300	68.03	5,525	69.60
Bihar	2,128	32.07	6,339	30.40

SC/ST: Scheduled caste/ scheduled tribe

Mean and standard deviation of depressive symptoms among adolescent male and female presented in the Table 2. Mean of the depressive symptoms was 1.0152 (SD = 0.1464) and 1.0797 (SD = 0.3416) among both male and female adolescents respectively. Higher the mean of depressive symptoms (Male: Mean = 1.0270; SD = 0.1629, Female: Means = 1.0869; SD = 0.3603) were among both illiterate males and females. Mean of the depressive symptoms was higher among those who had access to internet. Higher mean of the depressive symptoms was observed among females who used substances (Mean = 1.1072; SD = 0.4021) as well as among those who witnessed inter-parental violence (Mean = 1.0881; SD = 0.3488).

**Table 2:** Mean and SD of depressive symptoms by background variables

Background variables	Male		Female		t-test statistics
	Mean	SD	Mean	SD	p-value
Age group					
Adolescents (12–19 years)	1.0152	0.1464	1.0797	0.3416	< 0.001
Young adults (20–23 years)	1.0000	0.0000	1.0098	0.0988	0.009
**Educational level**					
Never	1.0270	0.1629	1.0869	0.3603	0.082
Primary &middle	1.0099	0.1280	1.0740	0.3305	< 0.001
Higher	1.0139	0.1369	1.0757	0.3313	< 0.001
**Substances use**					
No	1.0107	0.1258	1.0755	0.3321	< 0.001
Yes	1.0178	0.1529	1.1072	0.4021	< 0.001
**Inter-parental violence**					
No	1.0127	0.1333	1.0760	0.3343	< 0.001
Yes	1.0163	0.1560	1.0881	0.3488	0.002
**Internet access**					
No	1.0076	0.0987	1.0768	0.3360	< 0.001
Yes	1.0143	0.1426	1.0766	0.3339	< 0.001
**Paid work**					
No	1.0098	0.1167	1.0755	0.3323	< 0.001
Yes	1.0170	0.1557	1.0813	0.3455	< 0.001
**Mother’s education**					
No	1.0115	0.1301	1.0780	0.3396	< 0.001
Yes	1.0155	0.1428	1.0733	0.3231	< 0.001
**Wealth index**					
Poor	1.0090	0.1196	1.0736	0.3283	< 0.001
Middle	1.0132	0.1479	1.0671	0.3130	< 0.001
Rich	1.0145	0.1360	1.0825	0.3475	< 0.001
**Caste**					
SC/ST	1.0120	0.1318	1.0842	0.3439	< 0.001
Other	1.0132	0.1356	1.0743	0.3322	< 0.001
**Religion**					
Hindu	1.0115	0.1253	1.0737	0.3274	< 0.001
Non-Hindu	1.0200	0.1764	1.0878	0.3617	< 0.001
**Place of residence**					
Urban	1.0126	0.1321	1.0872	0.3609	< 0.001
Rural	1.0131	0.1367	1.0693	0.3153	< 0.001
**State**					
Uttar Pradesh	1.0178	0.1619	1.0748	0.3313	< 0.001
Bihar	1.0075	0.0967	1.0784	0.3385	< 0.001

SC/ST: Scheduled caste/ scheduled tribe

Bivariate correlations between outcome and key explanatory variables are presented in Table 3. Adolescents and young adults being victims of cyber-bullying [β = 0.1357, p < 0.001] or those witnessed inter-parental violence were positively [β = 0.0026, p < 0.001] correlated with depressive symptoms. Whereas, self-efficacy and adolescents parent communication were negatively related to depressive symptoms among adolescents and young adults.

**Table 3:** Bivariate correlations between outcome and key explanatory variables

Variables	Depressive symptoms	Cyber victimization	Self-efficacy	Parental communication	Inter-parental violence	Age	sex
**Depressive symptoms**	1						
**Cyber victimization**	0.1357***	1					
**Self-efficacy**	-0.1061***	0.0410***	1				
**Parental communication**	-0.0906***	0.0605***	0.1989***	1			
**Inter-parental violence**	0.0026	-0.0084	-0.0347***	0.043***	1		
**Age group**	0.1696***	0.0502***	-0.0009	-0.3493***	-0.145***	1	
**sex**	0.1932***	0.0482***	-0.2251***	-0.0617***	0.004	0.2956***	1

Note: ***: 95% level of significance

**Fig. 1:**
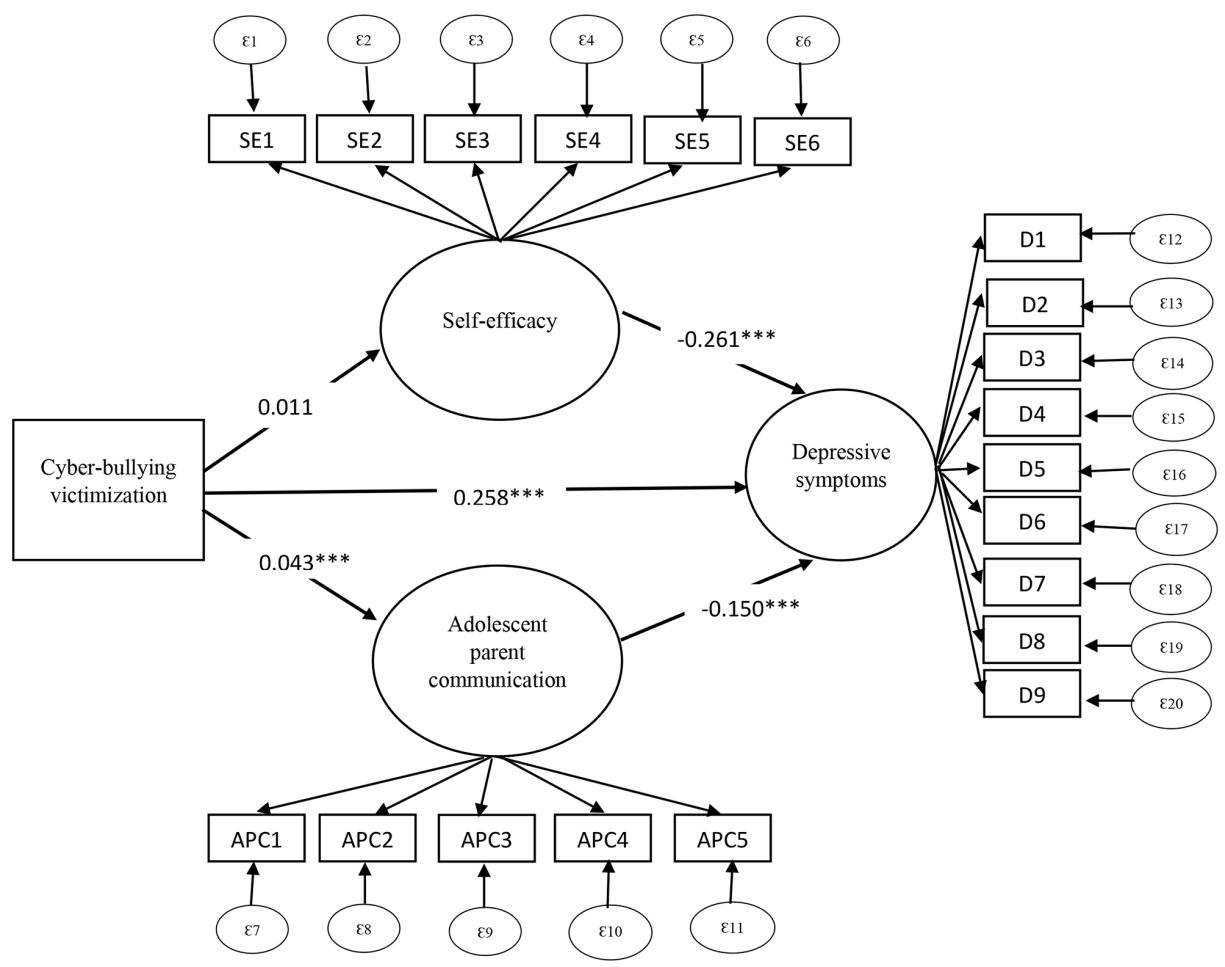
Standardized parameter estimates (βcoefficients) of the structural equation model (refer to Table 4 for the label name). Latent and observed variables are in oval and rectangular shapes, respectively Note: AC1: Discussed school performance, AC2:Discussed about friendship, AC3: Discussed about teasing, AC4: Discussed about physical changes, AC5: Discussed about pregnancy, D1: Trouble in sleep, D2: Feeling tired, D3: Poor appetite, D4: Trouble in concentration, D5: Little interest in doing things, D6: Feeling down, D7: Feeling bad, D8: Moving or speaking slowly, SE1: Sure about spending own time, SE2: Sure about participation in family/non-school related events/functions, SE3: Sure about earn income, SE4: Sure about talking freely to parents/in-laws about your aspirations, SE5: Sure about could choose clothing ***: 95% level of significance; Direct effect = 0.258; Indirect effect (via self-efficacy) = 0.011* (-0.261) =-0.00287; Indirect effect (via parental communication) = 0.043*(-0.150) =-0.00645

Figure 1; Table 4 indicate the generated model with the standardized parameter estimates. Since chi-square is sensitive to large sample sizes and always rejects the proposed model, the RMSEA, which is not sensitive to sample size, is considered. The RMSEA in this study indicated a good fit (RMSEA = 0.046), it was closer to the acceptable traditional level of 0.05.

**Table 4:** Multivariate regression coefficients (β), standard error (SE), and 95% confidence interval (CI) of the estimated structural equation model

Variables	β (SE)	95%CI
Adolescent’s parent communication		
Cyber victimization	0.0115(0.01)	(0-0.03)
Age (in years)	-0.0002(0)	(0–0)
Sex	-0.0945(0.01)***	(-0.11–0.08)
Secondary education	0.0406(0.01)***	(0.03–0.05)
Higher	0.1186(0.01)***	(0.1–0.13)
Internet access	0.075(0.01)***	(0.07–0.08)
Paid work	0.0612(0)***	(0.05–0.07)
Mother’s education	0.0309(0)***	(0.02–0.04)
Middle	0.0049(0.01)	(-0.01-0.02)
Rich	0.008(0.01)	(0-0.02)
Inter-parental violence	-0.0209(0.01)**	(-0.04-0)
caste	-0.0029(0)	(-0.01-0.01)
Urban	0.0101(0)**	(0-0.02)
Uttar Pradesh	-0.0028(0)	(-0.01-0)
**Self-efficacy**		
Cyber victimization	0.0434(0.01)***	(0.03–0.06)
Age (in years)	-0.0672(0)***	(-0.07–0.06)
Sex	0.0919(0.01)***	(0.08–0.1)
Secondary education	0.0174(0.01)*	(0-0.04)
Higher	0.2096(0.01)***	(0.19–0.23)
Internet access	0.0741(0.01)***	(0.06–0.09)
Paid work	0.0388(0.01)***	(0.03–0.05)
Mother’s education	0.1125(0.01)***	(0.1–0.12)
Middle	0.0202(0.01)***	(0.01–0.03)
Rich	0.0588(0.01)***	(0.05–0.07)
Inter-parental violence	0.0115(0.01)	(-0.01-0.03)
caste	-0.0149(0.01)**	(-0.03-0)
Urban	0.0534(0.01)***	(0.04–0.06)
Uttar Pradesh	0.0667(0)***	(0.06–0.08)
**Depressive symptoms**		
Parental communication	-0.2619(0.02)***	(-0.31–0.22)
Self-efficacy	-0.1501(0.01)***	(-0.18–0.12)
Cyber victimization	0.2581(0.01)***	(0.23–0.29)
Model fit statistics		
Chi-Square	0.000	
RMSEA	0.046	
CFI	0.821	
TLI	0.800	
SRMR	0.039	
CD	0.569	

Note: CD: Critical dimension, CI: Confidence interval, CFI: comparative fit index, RMSEA: root mean square error of approximation, SRMR: Standardized root mean square residual, TLI: Tucker-Lewis index, β (SE): regression coefficients (standard error), ***: 95% level of significance

### Measurement variables for depressive symptoms

All the measurement variables in the endogenous variables of depressive symptoms contributed considerably to the model and were statistically significant at P < 0.001.

### Multivariate structural model analysis

In the depressive symptoms pathway, there were significant linkages between cyber victimization and self-efficacy and parental communication. There was a positive relationship between cyber victimization [β = 0.258, p < 0.001] and depressive symptoms. Cyber victimization was positively and significantly related to self-efficacy [β = 0.043, p < 0.001] among adolescents and young adults. Self-efficacy [β= -0.150, p < 0.001] and parental communication [β=-0.261, p < 0.001] reduced depressive symptoms among the participants.

## Discussion

The current study explored the association between cyber victimization and the depressive symptoms of young adults and examined the role of self-efficacy and parental communication in the association by utilizing the structural equation model. The study used the self-efficacy scale and parental communication measures to bring empirical evidence of their effects on the relationship between cyber victimization and adolescents’ young adults’ depression and found significant mediation effects of self-efficacy and parental communication.

Previous studies reported an independent association between cyber victimization and the mental health of adolescents and young adults [25]. As expected, our findings demonstrated a positive association between cyber-bullying and depressive symptoms. However, self-efficacy and parental communication mediated the relation between these variables. In other words, with cyber victimization, lower level of self-efficacy and parental communication result into high level of depressive symptoms, but the high-level occurrence of these mediating variables reduce the probability of experiencing depression.

Many longitudinal studies discovered that prior cyber victimization predicted depression symptoms and suicidal ideation among adolescents throughout the follow-up period [1, 32, 33]. Previous research has indicated that cyber victimization raised the probability of developing negative traits including loneliness and a sense of helplessness, which elevated an individual’s risk for depression and suicide ideation [34]. Some literatures also claimed that a vicious circle develops between mental illness and cyber victimization. Adolescents who experience depression or have poor mental health may be more likely to use social media and the internet to mask or deflect their emotions. As a result, they are exposed to and more likely to experience cyber-bullying, which increases their risk of developing severe depression and suicide ideation [25, 35, 36].

These findings are consistent with other research on examining the involvement of adolescents in cyber-bullying episodes, and effects of inter-parental violence. Adolescents, having experienced inter-parental violence and cyber victimization combinedly are more prone to have depressive symptoms [17, 37, 38]. Higher levels of aggressive, depressive and anxiety symptoms were linked to higher levels of inter-parental conflict. And lower levels parental communication even worsens the situation. Communication with parents should be interpreted as a proxy for a number of contextual elements that may support and protect adolescent health. Regular family contact, which is facilitated by a more frequent discussion about personal matters or academic performance, allows for open dialogue between parents and children as well as an opportunity for adolescents to express difficulties and concerns as they develop. Therefore, it may support adolescents mentally and reduce the effects of stressful conditions [39]. Another study found that cyber-bullying victims are also tend to retaliate the act on their bullies as an act of revenge [40].

Self-efficacy is a notion that refers to how a person views themselves and their confidence in their own capacity to plan and carry out specific actions successfully in order to achieve the intended outcome. The relationship between self-efficacy and reporting of cyber victimization was significant in this study which is also observed in previous studies [41]. Cyber victimization and events were found to be strongly correlated with self-efficacy because the victims had a low sense of self-efficacy and thought they couldn’t handle the issue [42]. According to the social cognitive theory, people act as a result of their self-efficacy beliefs. Their preferred behavioral pattern and acquired skills are influenced by their self-efficacy beliefs. Levels of self-efficacy can influence healthier behavior. Because of this, those who have high degrees of self-efficacy in their ability to deal with cyber victimization successfully choose professional behavior. High self-efficacy people can handle difficult social settings and favor trusting behavior as a social tactic [43]. Additionally, it has been shown that people with high social self-efficacy receive more help from their friends when they are the victims of cyber-bullying, indicating the protective function of self-efficacy [44].

Nevertheless, the findings of our study have several important public health implications because the analyses are conducted in a large sample of young adults derived from a state-representative evidence-based dataset, which explored the effects of cyber-bully on depression of young population. These findings have implications for welfare practitioners and policy makers. In the light of the findings, it may be recommended to revisit the legalities, several academic institutions have anti-bullying policies that, in most cases, outline the penalties or disciplinary measures that will be taken against bullies, but legal remedies are not frequently mentioned. In such cases we recommend to reframe the policies in terms of cybercrimes. Taking decisive actions to improve mental health awareness through enhancing the peer connections and strengthening parental relationships among young adults would help them overcoming the depressive thoughts caused by cyber victimization. There is also a need for developing a specific professional training program with close supervision and guidance. It is also crucial to build new knowledge-based policies for young adults and their parents to educate them about the nature of nature of cyber-bullying and the primary coping strategies [45].

There are a number of inadequacies in the current study that underscore the need for future research. As our study relies on self-reported data, the method for measuring cyber victimization is indefinite and subject to both under- and over-reporting of cyber victimization. The fact that our study participants were in good health and that our results might not apply to victims of cyber-bullying who have been diagnosed with post-traumatic stress disorder should also be cautioned while interpreting the results. Because our data is based on an offline survey that was only conducted in two states, it is possible that the findings cannot be applied to all populations in the country. To minimize such biases, it is advised that future research include online surveys as well.

## Conclusions

The findings suggest that adolescents and young adults who are victims of cyber-bully may have depressive symptoms and their mental health can be improved through the enhancement of self-efficacy and increased parental communication. It is advised to the program designers and practitioners to put an emphasis on social factors associated with cyber victimization and develop intervention programs, such as social and familial support that may lessen feelings of loneliness, and devise strategies to raise young adults’ subjective well-being and perceived self-efficacy. Improved peer attitudes and familial support for empowering cyber victims should be taken into account while framing programs and interventions.

## Electronic supplementary material

Below is the link to the electronic supplementary material.

Supplementary Material 1 Table S1: Multivariate parameter estimates (β), standard error (SE) and 95% confidence interval (CI) of the measurement variables in the structural equation model

## Data Availability

The data that support the findings of this study are available from official site of project UDAYA by Population Council, and with the link: https://dataverse.harvard.edu/dataset.xhtml?persistentId=doi:10.7910/DVN/ZJPKW5, but restrictions apply to the availability of these data, which were used under license for the current study, and so are not publicly available. Data are however available from the authors [Chanda Maurya (Corresponding author)] upon reasonable request and with permission of Population council.
