# Supplementary material for: The role of self-efficacy and parental communication in the association between cyber victimization and depression among adolescents and young adults: a structural equation model
Source: BMC Psychiatry. 2023 May 12;23:337. doi: 10.1186/s12888-023-04841-6 (PMC10176763; doi:10.1186/s12888-023-04841-6)
Supplement: Supplementary file 1 — Supplementary Material 1 Table S1: Multivariate parameter estimates (β), standard error (SE) and 95% confidence interval (CI) of the measurement variables in the structural equation model [file 12888_2023_4841_MOESM1_ESM.docx]

**Table S1: Multivariate parameter estimates (β), standard error (SE) and 95% confidence interval (CI) of the measurement variables in the structural equation model**

| **Codes** | **Indicators** | | **Β(SE)** | **95%CI** |
| --- | --- | --- | --- | --- |
| **Self-efficacy** |  | |  |  |
| A1 | Could choose how to spend your free time? | | 1 |  |
| A2 | Could participate in no-family/non-school related events/functions? | | 3.1(0.11)*** | (2.89-3.31) |
| A3 | Can choose to earn an income if you wish to? | | 3.29(0.12)*** | (3.06-3.51) |
| A4 | Can talk freely to your parents/in-laws about their aspirations? | | 2.59(0.09)*** | (2.41-2.77) |
| A5 | Can choose the type of clothing that you would like to wear? | | 2.64(0.09)*** | (2.46-2.83) |
| A6 | Can play strong role in resolving family conflicts? | | 2.4(0.09)*** | (2.22-2.58) |
| **Adolescent parent communication** | |  |  |  |
| B1 | | Discussed about school performance with your mother or father? | 1 |  |
| B2 | | Discussed about your friendship with your mother or father? | 1.13(0.02)*** | (1.1-1.16) |
| B3 | | Discussed about being teased with your mother or father? | 0.41(0.01)*** | (0.39-0.43) |
| B4 | | Discussed about menstruation with your mother or father? | 0.78(0.01)*** | (0.76-0.81) |
| B5 | | Discussed about how pregnancy occurs with your mother or father? | 0.06(0)*** | (0.05-0.06) |
| **Depressive symptoms** | |  |  |  |
| C1 | | Had trouble falling asleep? Or  have you been sleeping too much? | 1 |  |
| C2 | | Feeling tired or having little energy like not feeling like talking to anyone, doing anything, going anywhere? | 1.12(0.02)*** | (1.08-1.16) |
| C3 | | Have you been had poor appetite?  have you been overeating? | 1.11(0.02)*** | (1.07-1.15) |
| C4 | | Trouble concentrating on things? | 1.12(0.02)*** | (1.08-1.16) |
| C5 | | Have you had little interest or pleasure in doing things | 1.08(0.02)*** | (1.04-1.12) |
| C6 | | Have you been feeling down, depressed or hopeless? | 1.3(0.02)*** | (1.26-1.34) |
| C7 | | Have you been feeling bad about yourself- or that you are a failure or have let yourself or your family down? | 0.93(0.02)*** | (0.9-0.96) |
| C8 | | Have you been moving or speaking so slowly that other people could have noticed? Or  being so fidgety or restless that you have been moving around a lot more than usual? | 0.77(0.01)*** | (0.74-0.8) |
| C9 | | Thoughts that you would be better off dead, or of hurting yourself in some way? | 0.38(0.01)*** | (0.37-0.4) |
| Var (e.A1) | |  | 0.5(0.01) | (0.49-0.51) |
| Var (e.A2) | |  | 1.08(0.01) | (1.05-1.11) |
| Var (e.A3) | |  | 0.92(0.01) | (0.9-0.95) |
| Var (e.A4) | |  | 0.88(0.01) | (0.86-0.91) |
| Var (e.A5) | |  | 0.8(0.01) | (0.78-0.82) |
| Var (e.A6) | |  | 1.15(0.01) | (1.13-1.18) |
| Var (e.B1) | |  | 0.12(0) | (0.11-0.12) |
| Var (e.B2) | |  | 0.1(0) | (0.1-0.11) |
| Var (e.B3) | |  | 0.08(0) | (0.08-0.08) |
| Var (e.B4) | |  | 0.13(0) | (0.13-0.13) |
| Var (e.B5) | |  | 0.01(0) | (0.01-0.01) |
| Var (e.C1) | |  | 0.5(0.01) | (0.49-0.51) |
| Var (e.C2) | |  | 0.47(0.01) | (0.46-0.48) |
| Var (e.C3) | |  | 0.55(0.01) | (0.54-0.56) |
| Var (e.C4) | |  | 0.3(0) | (0.3-0.31) |
| Var (e.C5) | |  | 0.26(0) | (0.26-0.27) |
| Var (e.C6) | |  | 0.35(0) | (0.34-0.36) |
| Var (e.C7) | |  | 0.28(0) | (0.27-0.28) |
| Var (e.C8) | |  | 0.2(0) | (0.19-0.2) |
| Var (e.C9) | |  | 0.14(0) | (0.14-0.14) |
| Var (e.Self-efficacy) | |  | 0.03(0) | (0.03-0.04) |
| Var (e.Adolescents parent communication) | |  | 0.06(0) | (0.05-0.06) |
| Var (e.Cyberbullying victimization) | |  | 0.18(0.01) | (0.17-0.19) |
